# Creosote in Treatment of Phthisis

**Published:** 1891-01

**Authors:** C. E Murphey

**Affiliations:** Atlanta, Ga.


### CREOSOTE IN TREATMENT OF PHTHISIS.

BY C. E. MURPHEY, M. D., ATLANTA, GA.

There is no disease which has had a greater number of remedies suggested for its cure and a larger number of deaths than that of phthisis. But at the present day medical opinion is largely in favor of the use of beechwood creosote as the leading remedy to alleviate this dreaded disease. Much attention and study is now paid to microbes, and it is generally believed that by the use of suitable germicidal drugs, the disease can be at least greatly benefited.

For the past few years I have used the creosote treatment in a number of cases, with more or less benefit in each case. The appetite improves, the cough is alleviated to great extent, the quantity of sputum diminished, the râles lessened, and the general health of the patient greatly improved.

In all cases I see that my patients are nourished and well provided with suitable clothing, such food as milk, eggs, rice, milk punches, egg-nog, etc.

The dosage and administration of the creosote is important. I use it both by inhalation and stomach or bowels. I have successfully employed, for the past two years in my practice, the following methods: The inhalation method naturally can be used to better advantage in patients whose gastro-intestinal tract is affected, while better satisfaction can be obtained by the stomach or bowels where a patient's digestive organs are in a fair condition.

For inhalation I have made a cone-shaped, tin funnel, closed at the top and the base, made to fit over the mouth and nose ; near the base I have a number of perforations for the air to pass out.

The solution used for inhalation is dropped on a soft sponge and the sponge placed at the bottom of the funnel. The combination used is five drops of creosote, ten drops of chloroform and twenty drops of alcohol; in addition I often use a small quantity of tincture iodine. As a patient becomes accustomed to its use I increase the creosote.

The inhalation should be full and deep, for ten or fifteen minutes at a time, four or five times daily.

We not only get full exercise of the lungs, but introduce, with the air, the creosote and iodine, of which favorable action on the mucous membrane and tubercle bacilli has often been proven. The chloroform has such quieting effect on the exhausting cough, that the patient never fails to carry his apparatus to bed with him and use it during the night if necessary. This method does not injure digestion as does the internal use of creosote. But in grave cases, and where the patient will stand the treatment, I prefer its use by mouth, rectum and inhalation simultaneously, in fact most rapid progress seems to be made when you utilize all three methods. The dose will be determined according to the quantity each individual will bear. The average patient will not tolerate more than eight to ten drops per day for any great length of time, and many others will only bear from three to five drops daily. Usually the best mode is to start with small dose and gradually increase the quantity to tolerance of the patient. It is important that the treatment should be uniform and regular, therefore if intolerance of the drug is shown by your patient in one method of treatment, then try another so that you can continue the treatment.

You will have to combine with suitable vehicles when you give creosote by the stomach, also give it in a quantity of water.

I generally employ the following formula:

R. Creosoti (beechwood), ʒss.

Pulv. acaciæ, ʒj.

Glycerini, ℥j.

Olii cinnamomi, gtt. ij.

Spt. frumenti, ℥j.

Aquæ puræ, q. s. ad. ℥iv.

Mix. Sig.—Teaspoonful in a fourth of a glass of water every three hours and gradually increase to two teaspoonfuls.

R. Creosoti (beechwood), ʒjss.

Balsam peru., ʒij.

Mucilag. acaciæ, ℥j.

Spt. frumenti, ℥ij.

Glycerini, q. s. ad. ℥iv.

Sig.—Teaspoonful in fourth of a glass of water after meals and gradually increase to two teaspoonfuls.

R. Creosoti (beechwood), ʒij.

Mucilag. acaciæ, ℥j.

Aquæ, q. s. ad. ℥iv.

Sig.—Teaspoonful in wineglass of sweet milk after meals.

By the bowel I use the following formulæ:

R. Creosoti (beechwood), ʒiijss.

Mucilag. acaciæ, ℥ij.

Tinct. opii., ℥ss.

Aquæ, q. s. ad. ℥iv.

Sig.—Teaspoonful in two ounces of tepid water; inject in bowels three or four times daily.

R. Creosoti (beechwood), ʒiijss.

Mucilag. acaciæ, ℥ij.

Aquæ, q. s. ad. ℥iv.

Sig.—Teaspoonful in two or three ounces of sweet milk; inject in bowels three or four times a day.

For injection use a three ounce hard rubber syringe. I do not claim creosote will cure consumption, but do claim the beneficial results in most cases will be surprising, and trust that the physicians generally will give creosote a trial and report the results.

